# Successful Transcatheter Edge‐To‐Edge Repair for Chronic Mitral Regurgitation Following Papillary Muscle Rupture

**DOI:** 10.1002/ccr3.71198

**Published:** 2025-10-06

**Authors:** Suguru Hirose, Manami Watahiki, Ikuko Shibasaki, Shigeru Toyoda

**Affiliations:** ^1^ Department of Cardiovascular Medicine Dokkyo Medical University Hospital Mibu Tochigi Japan; ^2^ Department of Cardiac and Vascular Surgery Dokkyo Medical University Hospital Mibu Tochigi Japan

**Keywords:** mitral regurgitation, myocardial infarction, papillary muscle rupture, transcatheter edge‐to‐edge mitral valve repair

## Abstract

Papillary muscle rupture (PMR) is a rare myocardial infarction complication, often associated with high surgical risk. This report highlights transcatheter edge‐to‐edge mitral valve repair as a feasible and less invasive alternative for high‐risk patients with chronic PMR.

## 1 Case Description

Papillary muscle rupture (PMR) is a rare but severe complication of acute myocardial infarction (AMI), occurring in approximately 0.03% of cases [1]. It frequently necessitates emergency surgery, with surgical repair remaining the standard therapy. However, the 30‐day mortality rate following surgery approaches 40% [2], particularly in older, frail, or hemodynamically unstable patients. In such high‐risk cases, transcatheter edge‐to‐edge mitral valve repair (M‐TEER) has emerged as a less invasive alternative, although reports in PMR are scarce and evidence remains limited [3].

An 84‐year‐old woman with a history of cerebral infarction and hypertension presented with ST‐elevation AMI (leads V2–V4). She initially declined percutaneous coronary intervention (PCI) and was discharged in stable condition after conservative management. One month later, she was readmitted to another institution with worsening heart failure. Transthoracic echocardiography revealed severe mitral regurgitation (MR) due to anterior PMR and triple‐vessel disease on coronary angiography. Although surgical mitral valve replacement and coronary artery bypass grafting are standard treatments, she was considered high‐risk, with a Clinical Frailty Scale score of 6 and a Society of Thoracic Surgeons predicted mortality of 16.2%. She again declined surgery. PCI achieved complete revascularization with one drug‐eluting stent each in the left anterior descending (#7), right coronary (#1), and left circumflex (#11) arteries. Despite this, she continued to present with New York Heart Association (NYHA) class III symptoms and was subsequently referred to our institution.

**FIGURE 1:**
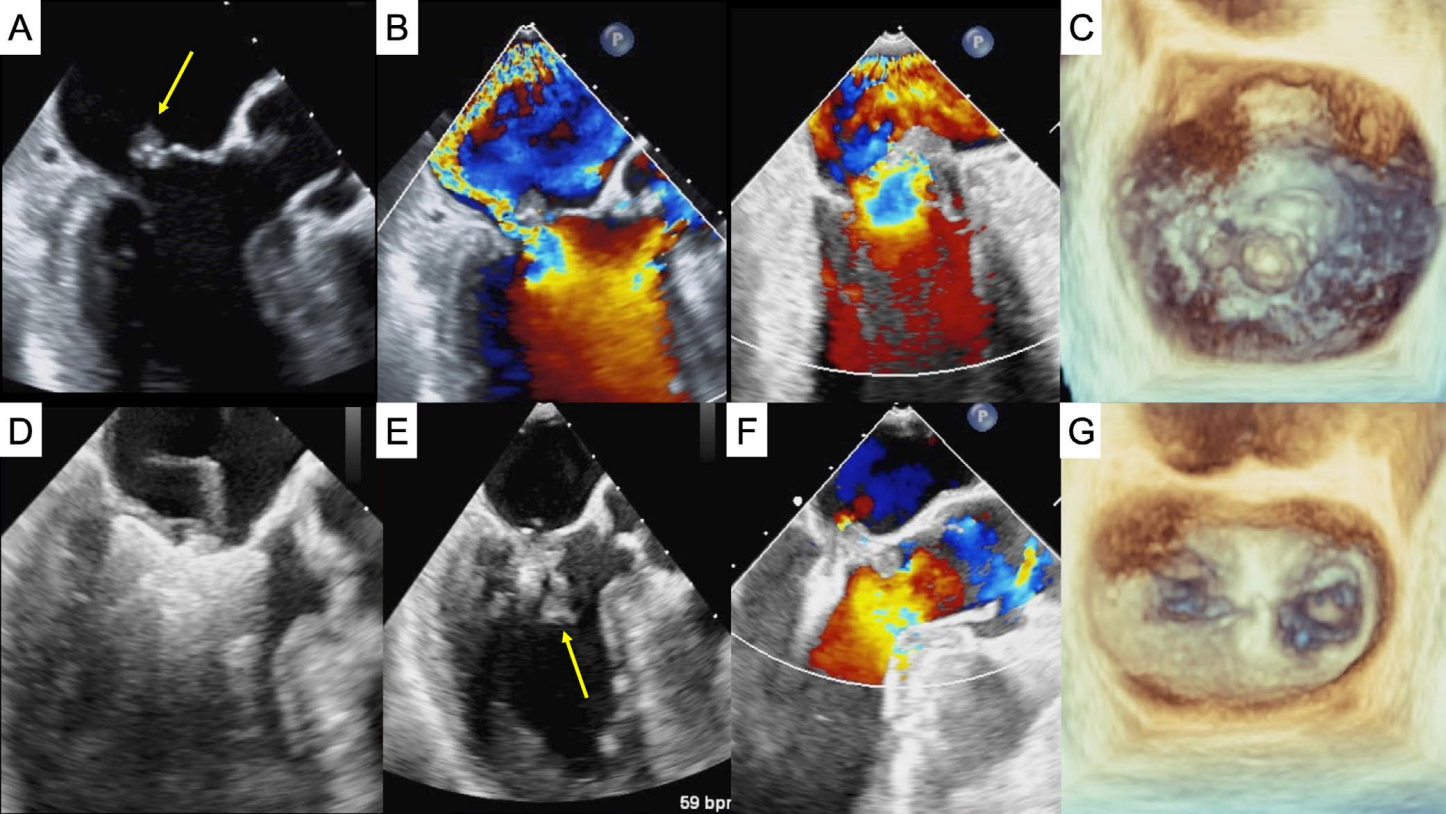
Transcatheter edge‐to‐edge mitral valve repair for papillary muscle rupture. (A) Rupture of the anterior papillary muscle (arrow). (B, C) Preprocedural TEE images, including left ventricular outflow tract views, bicommissural views, and three‐dimensional reconstructions. (D) Both mitral leaflets are securely grasped and deeply inserted into the clip arms. (E) The ruptured papillary muscle (arrow) is visualized within the left ventricle. (F) Postprocedural TEE shows trivial residual mitral regurgitation. (G) Three‐dimensional TEE image demonstrating a single clip creating a tissue bridge. TEE, transesophageal echocardiography.

Transesophageal echocardiography demonstrated partial anterior papillary muscle rupture, involving the A2 leaflet with measurable flail width of 8.4 mm and gap of 5.7 mm (Figure 1A–C). Right heart catheterization revealed no remarkable abnormalities (mean pulmonary capillary wedge pressure of 9 mmHg, mean pulmonary pressure of 20 mmHg). Given her surgical risk and preference for less invasive treatment, we opted for M‐TEER. The procedure was performed using the MitraClip G4‐XTW system (Abbott Vascular, California, USA), targeting the A2‐P2 segment. The mitral leaflets were securely grasped with deep insertion into the clip arms, and the ruptured papillary muscle was stabilized within the left ventricle during diastolic clip closure (Figure 1D,E; Video S1). Post‐procedure, the mean transmitral gradient was 3 mmHg and valve area was 2.3 cm^2^. MR was reduced to trivial, and the leaflet prolapse was resolved (Figure 1F,G). She was discharged in NYHA class I and remained asymptomatic at the 6‐month follow‐up, with only mild residual MR and no heart failure readmissions.

Surgical repair remains the standard treatment for acute PMR; however, many patients are inoperable due to hemodynamic instability or frailty. Conservative management is associated with poor outcomes, with a 2‐month survival rate of 6% [1]. Unlike the typical acute presentation, her chronic MR with stable hemodynamics enabled elective transcatheter intervention, achieving significant MR reduction with a single clip. M‐TEER may be a feasible alternative for patients with chronic PMR unsuitable for surgery. However, cases involving broad leaflet prolapse may require multiple clips, increasing procedural complexity and the risk of suboptimal MR reduction. Long‐term follow‐up is essential to further define the role of M‐TEER in this rare condition and optimize patient selection and procedural strategies.

## Author Contributions

**Suguru Hirose:** visualization, writing – original draft. **Manami Watahiki:** visualization. **Ikuko Shibasaki:** writing – review and editing. **Shigeru Toyoda:** writing – review and editing.

## Consent

Written informed consent was obtained from the patient for the publication of this case report and accompanying images, in accordance with the journal's patient consent policy.

## Conflicts of Interest

The authors declare no conflicts of interest.

## Supporting information


**Video S1:** Grasping of the mitral valve leaflets during transcatheter edge‐to‐edge mitral valve repair using the MitraClip system.

## Data Availability

All data are included in this article and Video S1.
